# Financial Toxicity in Radiation Oncology

**DOI:** 10.7759/cureus.58643

**Published:** 2024-04-20

**Authors:** Kanchan Gupta, Bhupesh Parashar

**Affiliations:** 1 Biology, Moravian Academy, Bethlehem, USA; 2 Radiation Oncology, Zucker School of Medicine at Hofstra/Northwell, Lake Success, USA

**Keywords:** economic hardship, cancer, radiation therapy, out of pocket costs, financial toxicity

## Abstract

Financial toxicity details the financial burden patients face due to a variety of medical costs. Cancer patients, especially those receiving radiation therapy, are at a much higher risk of experiencing economic hardships than healthy people or people with other conditions. There are a variety of risk factors associated with financial toxicity as well as numerous tools to assess the toxicity experienced by patients. In this review article, we present a concise overview of contributors, risk factors, case studies, tools, impacts, and potential interventions of financial toxicity.

## Introduction and background

Financial toxicity describes economic hardships faced by patients caused by their medical needs. There are numerous cancer-specific contributors that lead to high out-of-pocket costs and increased financial toxicity for patients including prescription drugs, impatient stays, emergency room visits, outpatient visits, and other medical needs [1]. Due to gaps in insurance coverage and high costs of care, financial toxicity may be experienced more in the USA than in other countries [2]. Other contributors include copayments, deductibles, and coinsurance [3].

## Review

Increased risk for cancer patients

Cancer patients have increased chances of experiencing financial toxicity than those without cancer and chronically ill individuals [4]. A total of 13.4% of patients with cancer from the 2001-2008 Medical Expenditure Panel Survey (MEPS) experienced financial burdens compared to 9.7% of patients with chronic conditions and 4.4% of patients with no significant medical issues [4]. Cancer survivors report spending more than 20% of their income on health care [1]. Consequently, national spending associated with cancer is already $200 billion (about $620 per person in the US) in the United States and continues to grow [1]. Cancer is a highly unpredictable illness with high risks of complications, relapses, and/or disease progression, all of which increase and sustain costs [1]. In addition, costs are increased through long-term therapies and unplanned visits due to acute symptoms of the disease and/or side effects from treatment [1]. At least 20% of all patients with cancer experience a level of financial toxicity severe enough to cause major changes in their day-to-day lives and health [5]. In addition, half of all cancer survivors continue to experience financial toxicity after treatment has ceased [1]. While all people with cancer are at risk for financial toxicity, studies show that nonelderly cancer patients in the following categories experience higher out-of-pocket costs: have private non-group insurance, age between 55 and 64, non-Hispanic, black, never married, never widowed, one or no children, unemployed, low income, low education level, reside in nonmetropolitan statistical areas or have other chronic conditions [4].

Risk factors

The level of financial toxicity experienced by an individual depends on several factors including how much money people in the same household earn, the amount of debt owed prior to diagnosis, assets, cost of cancer treatment (varies with diagnosis), ability to work after diagnosis, and insurance coverage [3].

There are a variety of risk factors associated with financial toxicity. Advanced-stage and/or metastatic cancer results not only in expensive treatments and constant monitoring but requires a specific treatment course focused on slowing the progression of the disease as opposed to eliminating it. These advanced treatments often require patients to spend most of their time in different care facilities. However, there are many uncertainties in terms of treatment due to the complexity and lack of uniform guidelines regarding metastatic cancer. Metastatic cancer is disproportionately diagnosed in ethnic and/or racial minorities and low-income individuals resulting in low survival rates and high risks for financial toxicity for this population. Research related to financial toxicity in metastatic cancer patients is difficult to conduct because each case is unique, and these patients are a minority in relation to all cancer patients [5]. Recurrent cancer, cancer with a poor prognosis, multiple types of cancer, and chronic disease in addition to cancer are also risk factors for increased financial toxicity [3]. Younger patients tend to have higher risks than older patients. Hardships are experienced twice as much by younger patients than older patients [1]. This is a result of a lack of savings, dependent children (more financial responsibilities), and a lack of health insurance, as insurance such as Medicare is only available after the age of 65 [1]. Both racial and ethnic minorities also experience increased risks of financial toxicity after considering education, income, and employment [6]. Low-income individuals are at higher risk due to working jobs with less flexibility, employee protection, and health benefits [5]. Location can increase the risk of financial toxicity for individuals as specialized care may not be available in some areas resulting in long transportation times and housing needs [1]. Both the job and marital status of an individual contribute to the level of financial toxicity they experience [7]. Based on a study done with patients with lung cancer in Western China, risk factors included young age, lower income, employed but on sick leave, and poor psychological status [8]. The length of treatment does not correlate to the severity of financial distress experienced by a patient; this means that patients are at risk of financial distress at any point during their treatment [9]. Women also have increased risks of financial toxicity for multiple reasons such as they make only 82% of their male counterparts’ wages. In addition, they are more commonly the primary caregiver for children and complete a disproportionate amount of unpaid housework [10].

Factors that should be considered include the ability of an individual to make ends meet at both the time of treatment and in the future, financial adjustment options, burden related to said adjustments, and bureaucracy [11].

Radiation oncology

The effect of radiation treatment on the financial toxicity experienced by cancer patients is difficult to quantify as there is no standardized way to measure the financial distress caused by treatment [12]. A 2020 study was completed using the Comprehensive Score for Financial Toxicity (COST) survey with a population of 167 patients [12]. On univariate analysis, patients actively completing radiation therapy (RT) and patients who had already completed RT significantly corresponded with lower COST scores (P = 0.03), indicating an increased effect on patient welfare [12]. The correlation between radiation oncology patients and lower COST scores indicates the increased risk of financial toxicity among these individuals.

An increase in hypofractionation is the most favorable way to reduce financial toxicity in radiation oncology [13]. Fewer appointments and/or shorter treatment courses provide numerous opportunities for patients to decrease spending and time away from work [13]. Hypofractionated regimens are being increasingly used both for curative as well as palliative treatments in brain malignancies, head and neck cancers, thoracic cancers, colorectal cancers, pancreatic cancers, gynecological cancers, skin cancers, and palliation, among others [14]. We discuss a few recent examples of the cost-effectiveness of hypofractionated short-course radiation.

In a retrospective study of 53 patients with soft tissue sarcomas treated with hypo-fractionated accelerated RT postoperatively, 32-35 standard fractions were replaced with 28 fractions [15]. In addition to 2-year local control of 100% and lower field size, there was a decrease in the estimated average treatment cost of $3056 (range, $2651-$4335; P <0.001). In a study evaluating Medicare and Medicaid services data in breast cancer (BC) and prostate cancer (PC) patients, median total spending for short-course RT regimens among BC episodes was $9418 versus $13,602 for long-course RT [16]. Among PC patients, median total spending was $6924 for hypofractionation RT, $18,768 for moderate hypofractionation, and $27,319 for long-course RT.  In a prospective randomized clinical trial including 222 patients from six French cancer centers was conducted as international PROstate Fractionated Irradiation Trial (PROFIT), a cost-effectiveness analysis (CEA) from the payer's perspective, showed that total costs per patient were lower in the short course arm compared to the long-course conventional arm €3,062 (95 % CI: 2,368 to 3,754) versus €4,285 (95 % CI: 3,355 to 5,215), (p < 0.05) [17]. Quality-adjusted life years were marginally higher in the short course arm, however, this difference was not significant: 0.044 (95 % CI: - 0.016 to 0.099).

Screening tools created for general oncology patients are not suitable for measuring financial toxicity in radiation oncology treatment patients as it does not account for elements specific to radiation oncology [18]. A 2021 study sought to create a radiation oncology-specific tool that identified early indications of financial toxicity with three variables: age, money owed, and worries related to copayments [18]. The 25-item comprehensive survey was given to 157 patients prior to treatment and successfully identified financial toxicity with the three variables stated above [18].

RT has been increasingly used as an ablative therapy in patients with metastatic cancer [19]. However, many of these patients still have short life expectancies [19]. Therefore, radiation oncologists should consider the utilization of RT in reference to its financial and physical implications as opposed to end-of-life care based on the patient’s individual diagnosis, goals, and the effectiveness of treatment [19]. Unnecessary treatment can result in extra costs and burdens for both the patient and their families/caregivers [19].

Case studies

Data from a 2010 nationally representative sample demonstrated that newly diagnosed cancer patients aged between 18 and 63 years old report an average of $1,107 in annual out-of-pocket costs. This is more than previously diagnosed cancer patients who report $747 in annual out-of-pocket costs. However, both groups have increased debts than those with no cancer history who report $617 in annual out-of-pocket costs [3].

A 2014 longitudinal study used population-based registries to study financial toxicity in long-term BC survivors. 3,133 women were surveyed approximately 9 months after their initial diagnosis and then 4 years later. All the women were diagnosed with nonmetastatic BC from 2005 to 2007. Eighteen percent of the women paid between $2000 and $5000 in out-of-pocket costs, while 17% paid more than $5000 in out-of-pocket costs. In terms of race, the number of individuals who reported having medical debt varied significantly: 9% of whites, 15% of blacks, 17% of English-speaking Latinas, and 10% of Spanish-speaking Latinas. This study statistically proved that racial and ethnic minorities are at greater risk of financial toxicity caused by a cancer diagnosis [6].

A 2015 study was conducted among 4,271 nonelderly cancer survivors using the 2008-2012 MEPS and evaluated its data using multivariable logistic regression. The study concluded that 4.3% of cancer survivors, aged between 18 and 64 years old, reported higher out-of-pocket medical costs than those without any history of cancer [20].

Tools to study financial toxicity

**Table 1:** Tools For Studying Financial Toxicity

Tools to Study Financial Toxicity	
Financial tool	Description
Comprehensive Score for Financial Toxicity (COST)	An 11-item survey that covers financial spending, financial resources, and the psychological response of an individual to assess/evaluate their level of financial toxicity Most widely used tool as it has been validated and demonstrates high internal consistency and test-retest reliability [21].
Breast Cancer Finances Survey (BCFS)	A 42-item tool that details individual components of cancer-related economic burdens such as psychosocial aspects, material hardships, coping methods, and out-of-pocket costs [22].
Personal Finance Wellness Scale (PFW scale)	Noncancer-specific scale designed primarily to focus on subjective distress related to finances Data from previously existing databases is frequently used to conduct research regarding financial toxicity.
Subjective Financial Distress Questionnaire (SFDQ)	A 17-item survey created to assess financial toxicity among radiation oncology patients showed adequate reliability and consistency among a small population but requires validity with a larger sample size [23].
Financial Index of Toxicity	A 9-item instrument was developed to assess financial toxicity in patients with head and neck cancer. Both its internal consistency and test-retest reliability were good; however, the study was limited to one facility and required samples from various facilities to validate its generalizability [24].

Impacts of Financial Toxicity

The impacts of financial toxicity are quite severe and affect numerous aspects of an individual’s life. Productivity loss has been proven to correlate with an increase in medical costs for both cancer survivors and patients [25,26]. This can result in material hardship such as lost wages and cost-related behavioral changes [5]. Cost-related behavioral changes include changes in medical spending such as declining or delaying care; changes in nonmedical spending include patients using money from savings to pay medical bills or not paying them at all [5]. Financial toxicity also leads to psychological burdens such as distress and/or anxiety [5]. Financial toxicity is associated with working-age cancer survivors experiencing a fear of cancer recurrence, loss of purpose, and loss of hope [27]. Data regarding financial toxicity is not routinely collected and is difficult to quantify, so few studies have reported financial toxicity among those with new diagnoses [18].

Potential interventions

Potential interventions for financial toxicity include focusing on the relationship between the oncologist and the patient. Oncologists should encourage patients to discuss costs and should be better educated on this matter so they can inform their patients of the financial resources available to them [28]. A 2023 study involving 233 chemoradiation patients indicated that patients were experiencing financial toxicity prior to starting treatment [29]. This demonstrates a need to both assess and manage financial toxicity prior to treatment as hospital visits for treatment generally increase the effects of financial toxicity on the patient’s quality of life [29].

## Conclusions

Financial toxicity poses a significant challenge for patients, especially those diagnosed with cancer, as they face high out-of-pocket costs and various medical needs contributing to economic hardships. Cancer patients, particularly those with advanced-stage or metastatic cancer, are at increased risk of experiencing financial toxicity, which can have lasting effects even after treatment ends. Addressing this problem requires a multi-faceted approach, including better communication between healthcare providers and patients, tailored interventions, and ongoing research to refine measurement tools.
